# A study of affecting the recovery of Chinese patients with Bell palsy

**DOI:** 10.1097/MD.0000000000014244

**Published:** 2019-01-25

**Authors:** Hongbo Zhang, Haixia Du, Mingjing Qian, Yu Wang, Shenghua Zhou, Jing Chen, Haitong Wan, Jiehong Yang

**Affiliations:** a Institute of Cardio-Cerebrovascular Diseases, Zhejiang Chinese Medical University, Hangzhou; b Department of Neurology, the First People's Hospital of Huzhou; c Department of Neurology, the Third People's Hospital of Huzhou, Zhejiang Province, Huzhou; d College of Basic Medical Science, Zhejiang Chinese Medical University, Hangzhou, China.

**Keywords:** Bell palsy, risk factors, therapeutic factors

## Abstract

We explored the risk factors for preventing recovery of Bell palsy (BP) in Chinese inpatients. Five hundred thirteen patients were included. The two end-points of assessment were the discharge and final follow-up results. Relationship between discharge and baseline: long patients delay (unhealed 4.03 ± 1.16 d vs improved 2.24 ± 1.0 d, *P* < .001), combined diseases (yes 77.06% vs no 86.71%, *P* = .01), and early use of acupuncture (yes 47.46% vs no 97.62%, *P* < .001) were bad factors. Therapeutic factors and discharge: only use of steroids was a positive factor (yes 92.54% vs no 57.30%, *P* < .001). Binary logistic regression found that early use of steroids was a favorable factor (*P* = .001), while early use of acupuncture (*P* < .001) and long patient delay (*P* < .001) were adverse factors. Subgroups analysis showed early use of steroids plus antivirals (steroids + antivirals vs antivirals + mecobalamin, *P* < .001) and early use of steroids plus mecobalamin were good choices (steroids + antivirals vs steroids + mecobalamin, *P* = .745), while early use of antivirals plus mecobalamin was a bad choice (vs other 2 groups, *P* < .001). Effect of drug dose and treatment course on discharge: long time use of steroids didn’t mean good efficacy (unhealed 10.80 ± 1.53 d vs improved 10.38 ± 1.21 d, *P* = .026). Final follow-up results: improved patients were better than that of unhealed at discharge (*P* < .001). Risk factors of discharge included long patient delay, combined diseases, and early use of acupuncture. Steroids plus antivirals or steroids plus mecobalamin were good choices for treatment. Long time use of steroids didn’t mean good effect. Improved patients at discharge had better results finally.

## 1 Introduction

Bell palsy (BP), an idiopathic peripheral nerve disorder that causes sudden paralysis of unilateral facial muscles, which was named by Sir Charles Bell (1774–1842) who described the syndrome along with the anatomy and function of the facial nerve.^[1]^ It is reported that BP is the most common disease of acute facial paralysis, and its incidence is 20 to 30 per 100,000.^[2]^ Peak incidence occurs in the fifth decade of human, but it may occur at any age, and the syndrome is more common in patients with diabetes and in pregnant women.^[3]^ The exact cause of the disease is still unclear, it is considered to be associated with viral infection.^[4]^

Most of the patients with BP have a good prognosis in clinical practice. Approximately 85% of patients with BP experience spontaneous recovery without treatment.^[5]^ But there are a few people especially with combined diseases such as diabetes still have a poor recovery.^[6]^ For therapeutic factors, the only definite recommendation is early use of steroids according to the guidelines of the 3 countries of the United States, China, and Canada, while antivirals are only recommended combined with steroids.^[7–11]^ It is also reported that the combination of antiviral drugs and steroids is less effective than use steroids alone.^[12]^

In clinical practice, we found that most Chinese doctors didn’t read related guidelines, so the treatment plans were scarcely under the guidance of guidelines. How do Chinese doctors deal with these patients? Is it consistent with these guidelines unconsciously? How many risk factors that affect the recovery of the patients? Is the traditional Chinese acupuncture therapy effective? It is a very important question whether good treatments for risk factors can be screened out to improve the efficacy. Thus, we analyzed 513 inpatients with BP in 5 years coming from 2 major Three Level Grade A hospitals in Huzhou, which located in one middle city of China, to understand the diagnosis and treatment of Chinese doctors.

## 2 Methods

### 2.1 Participants

**Figure 1:**
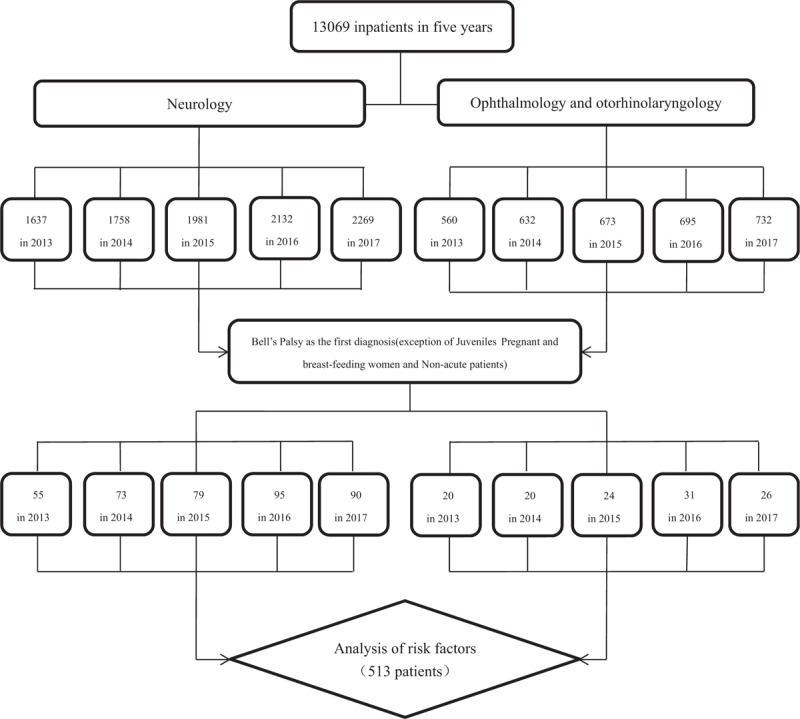
Study roadmap.

We conducted a retrospective analysis of hospitalized patients with BP from January 1, 2013 to December 31, 2017 (this was a retrospective observational study that didn’t involve ethical issues, and therefore didn’t require ethical approval). The patients came from 2 major Three Level Grade A hospitals (The First people's Hospital and the Third people's Hospital) of Huzhou, Zhejiang province, China. All clinical data were anonymized by the workers of the archives before any authors accessed them for the study. The study roadmap is shown in Fig. 1. Firstly, we retrieved the list of inpatients in the department of Neurology and Ophthalmology-otorhinolaryngology in 2 hospitals through the electronic medical records according to years (the inpatients number is 2192 in 2013, 2390 in 2014, 2654 in 2015, 2827 in 2016, and 3001 in 2017). Secondly, patients with first diagnosis of BP were screened out (the number was 86 in 2013, 105 in 2014, 118 in 2015, 143 in 2016, and 138 in 2017). Finally, pregnant and breast-feeding women, juveniles, and non-acute patients were excluded, 513 patients were eventually included in the study.

Table 1 shows the demographic characteristics and clinical details of 513 participants at baseline. All clinical items were assessed by an experienced neurologist. The clinical data included sex, age, patients delay days, combined diseases (in addition to BP as the first diagnosis, patients may have >2 diagnoses, but we only think that they have diabetes with combined diseases), and use of acupuncture or not before hospitalization. Many national guidelines, including China, do not recommend acupuncture as a way of treatment. So we suggested patients stopping the use of acupuncture in acute phase. For those patients insisted on using of acupuncture, the commonly used therapeutic acupoints include Cunzhu, Yangbai, Chengxie, Sibai, Dicang, Xiaguan, Cheekche, Hegu, etc. Frequency is once a day, lasting from 1 day to 2 weeks.

**Table 1:**
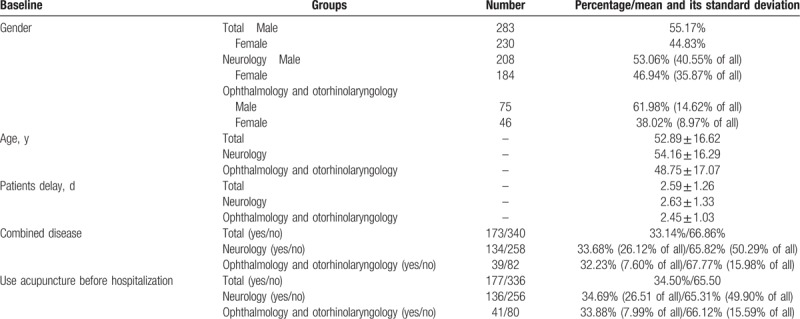
The demographic characteristics and clinical details of 513 inpatients at baseline.

### 2.2 Assessment of the patients with BP

We evaluated the patient's condition at admission, including the severity of BP and the combined diseases through the first disease process of the electronic medical record. Many studies looked upon 3 days as acute phase.^[13]^ We defined that no >10 days at admission were as the acute period and found out the patient's outcome at discharge through the discharge record and the first sheet of the medical record. We couldn’t use the related assessment scales to get the exact severity of the disease because it was a retrospective study,^[14,15]^ and the evaluation results we used were 2 options-improved and unhealed.

### 2.3 Procedure

First of all, we collected general information on the baseline of the selected patients. The use of drugs was comprehended by the physician order sheet. We also analyzed and compared the guidelines of the 3 countries (the United States, China, and Canada) about BP (Table 2). Finally, after 1 month, the telephone follow-up after discharge in all of the patients was conducted as part of routine clinical care and included in the medical records. While, with the aim to understand the recovery after discharge, about 20% patients were followed-up by telephone according to the discharge results after 3 months (this was a routine follow-up by telephone of the patients after discharge, without involving treatment, etc., and it didn’t need the authorization of the patients).

**Table 2:**
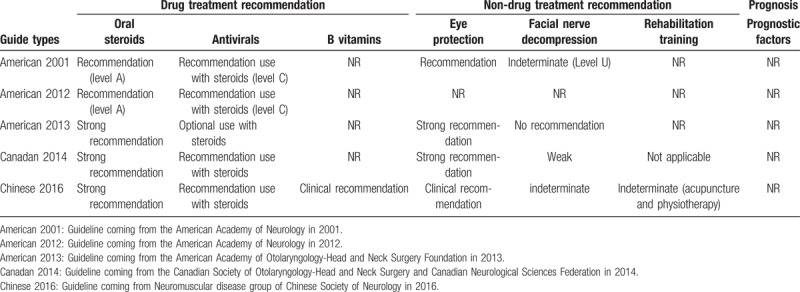
Guidelines comparison of China, America, and Canada about Bell palsy.

### 2.4 Statistical analysis

All analyses were performed using the Statistical Package for the Social Sciences version 22.0 for Windows. All continuous data, such as age, patients delay, and drug doses were presented as the mean ± standard deviation (SD). Student *t* test was used to compare continuous variables between patients improved and unhealed at discharge. The chi-square test was used to evaluate difference in categorical variables between patients improved and unhealed at discharged. A binary logistic regression model was used to explore potential factors related to discharge outcome. The improved and unhealed were the dependent variable, and independent variables included: age, sex, patients delay, combined diseases, early use of acupuncture, early use of steroids, early use of antivirals, and early use of mecobalamin. For subgroups, chi-square test using R × C unserial table firstly for all data, then using chi-square test to compare 2 different groups. All statistical tests were 2-tailed, and *P* < .05 was considered to be statistically significant (for multiple comparisons of the chi-square test, *P* < .0167 was considered to be statistically significant).

## 3 Results

### 3.1 Participants’ demographic characteristics and clinical items

A total of 590 Chinese patients with BP were recruited to examine the relationship between discharge outcome and related factors. Twenty-seven Juveniles, 9 pregnant and breast-feeding women, and 41 patients with delays of >10 days were excluded. Finally, 513 participants were included in the study. Of the 513 patients, 283 cases were men (55.17%), and 208 cases came from neurology (53.06% of neurology and 40.55% of all); 230 cases were women (44.83%), and 184 cases came from neurology (46.94% of neurology and 35.87% of all). Other patients came from mophthalmology and otorhinolaryngology. The average age and average delay days of the patients were 52.89 ± 16.62 and 2.59 ± 1.26, respectively. Table 1 showed more details of the variables.

### 3.2 Association between variables at baseline and outcome at discharge

Table 3 showed 234 (82.69% of men and 45.61% of all) inpatients of men improved when they were discharged from the hospitals, while 178 (77.39% of women and 34.70% of all) were women. There was no significant difference in the prevalence of improved between male and female patients (men 82.69% vs female 77.39%, *P* = .174). And age factors didn’t affect the prevalence of improvement (improved 52.27 ± 16.52 vs unhealed 55.41 ± 16.84, *P* = .089). However, the patients delay was one of the important factors of adverse consequences at discharge (improved 2.24 ± 1.01 d vs unhealed 4.03 ± 1.16 d, *P* < .001). The other 2 factors were combined disease and use of acupuncture, respectively. BP as a single disease was superior to combined diseases in outcome at discharge (Yes 77.06% vs No 86.71%, *P* = .010). Use of acupuncture before admission was a bad factor for improvement (Yes 47.46% vs No 97.62%, *P* < .001).

**Table 3:**
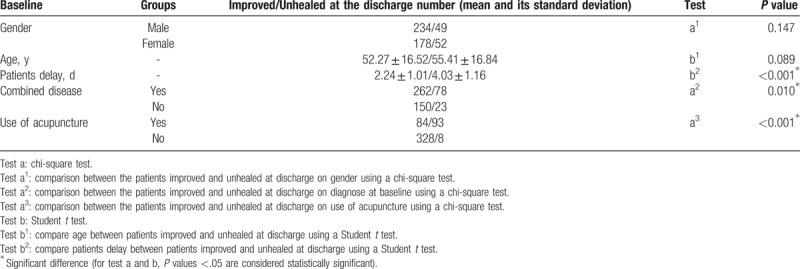
Association between variables at baseline and outcome at discharge.

Table 4 showed the relationship between the therapeutic factors and the outcome at the discharge. Only use of steroids was a positive factor for good outcome (Yes 92.54% vs No 57.30%, *P* < .001). And other variables including use of antivirals (yes 76.43% vs no 84.40%, *P* = .023), mecobalamin (yes 78.08% vs no 85.81%, *P* = .046), and acupuncture (yes 47.46% vs no 97.62%, *P* < .001) were negative factor for good outcome.

**Table 4:**
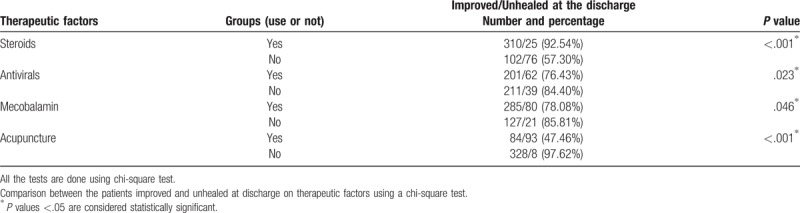
The relationship between the therapeutic factors and the outcome at the discharge.

We brought the relevant variables including baseline and therapeutic factors into a binary logistic regression model to explore the risk factors (Table 5). These variables not only included the factors that have statistical differences, but also included the incorporate factors such as age and sex to adjust. The results showed that the early use of steroids was a favorable factor (*P* = .001), while the early use of acupuncture (*P* < .001) and the long patient delay (*P* < .001) were adverse factors. Sex (*P* = .392), age (*P* = .382), combined diseases (*P* = .190), use of antivirals (*P* = .077), use of mecobalamin (*P* = .061) had no statistical differences.

**Table 5:**
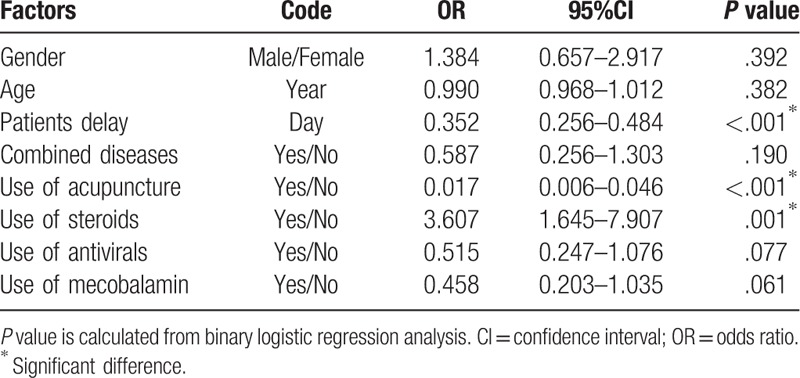
Associations between clinical factors and outcome at discharge.

After analyzing the guidelines of the 3 countries, early use of steroids was unanimously recommended, while use of antivirals was recommended combination with steroids (Table 2). Table 6 showed the analysis of 3 subgroups of combined treatment. Statistics demonstrated that the program of early use of steroids plus antivirals was indeed an optimized scheme (steroids + antivirals vs antivirals + mecobalamin, *P* < .001), and another program of early use of steroids plus mecobalamin was a good choice, too (steroids + antivirals vs steroids + mecobalamin, *P* = .745). But early use of antivirals plus mecobalamin was a bad choice (vs other 2 groups, *P* < .001).

**Table 6:**
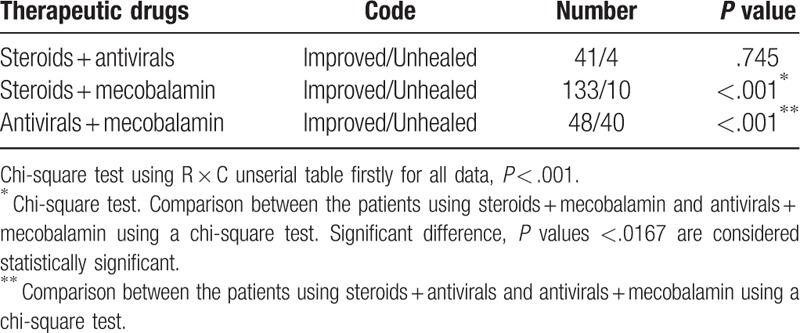
Association between the best treatment method based on guidelines and outcome at discharge.

**Figure 2:**
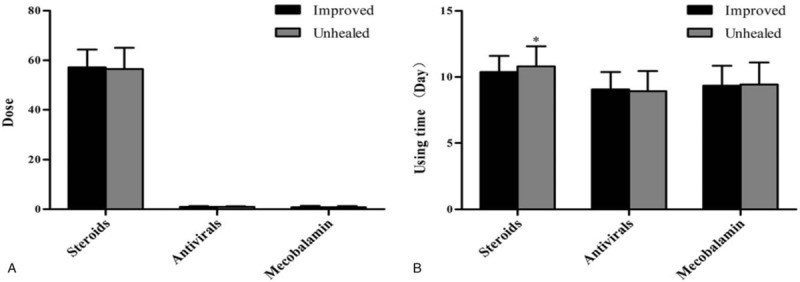
Associations between the dose of drugs (using time) and outcome at discharge. A. Dose of steroids, antivirals, and mecobalamin; B. Using time (day) of steroids, antivirals, and mecobalamin. Student *t* test. ^∗^*P* values <0.05 are considered statistically significant.

Figure 2 indicated the effect of drug dose and treatment course on the outcome. The result showed that long time use of steroids didn’t mean good effect (improved 10.38 ± 1.21 d vs unhealed 10.80 ± 1.53 d, *P* = .026). There were no statistical differences in other factors, such as steroids dose (improved 57.18 ± 7.14 mg vs unhealed 56.40 ± 8.60 mg, *P* = .264); antivirals dose (improved 0.99 ± 0.31 g vs unhealed 0.94 ± 0.30 g, *P* = .467), use time of antivirals (improved 9.05 ± 1.33 d vs unhealed 8.92 ± 1.52 d, *P* = .185); mecobalamin dose (improved 0.86 ± 0.48 mg vs unhealed 0.83 ± 0.47 mg, *P* = .250), use time of mecobalamin (improved 9.34 ± 1.52 d vs unhealed 9.43 ± 1.68 d, *P* = .196).

Table 7 showed the final outcome of the patients through telephone follow-up. The final outcome of improved patients at discharge was better than that of unhealed patients (*P* < .001).

**Table 7:**
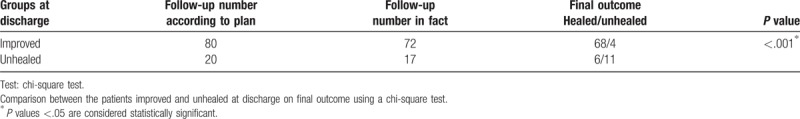
Relationship between discharge condition and final recovery.

## 4 Discussion

BP is a common disease which leads sudden unilateral facial paralysis in Chinese patients. According to the report, BP affects 20 to 30 persons per 100,000 annually, and 1 in 60 individuals will be affected over the course of their lifetime.^[16,17]^ Most of the prognosis is good, but a few can’t heal, leading to lifelong sequelae, such as synkinesis, hemifacial spasm, contractures, salivation, and so on.^[18]^ Although the unknown cause is an important reason for this poor curative effect, the risk factors are also very important. Panagiotis Kokotis and Serafeim Katsavos study found that the wind chill and temperature were risk factors of BP.^[19]^ Guidelines of the 3 countries did not give advises for controlling or treating risk factors. In the study, we found that unhealed inpatients at discharge had a bad outcome eventually. Therefore, it is very important to treat symptoms or other concurrent conditions during hospitalization and control previous diseases. In the acute phase (except for pregnant and breast-feeding women), age and sex are not risk factors in our study, which is consistent with another study.^[20]^

Our study found that the long patient delay, combined diseases, early use of acupuncture were detrimental to the patients’ recovery. Of the combined diseases, diabetes is a very important risk factor, which is consistent with other reports.^[21,22]^ Although Chinese doctors usually used the methods of oral administration of steroids and enhanced hypoglycemic treatment for these diabetic patients, they still have been observed to be inefficient in clinical practice. Some reports suggested that early acupuncture treatment was beneficial, while our results were just the opposite. Perhaps this is the reason why we suggested stopping the use of acupuncture in acute phase, and we indeed observed that many patients who insisted on using acupuncture eventually got bad results. This is a very controversial issue^[23–25]^ that requires further study of large scale samples to prove whether it is valid.

As for drug treatment, only use of steroids has sufficient evidence to be effective. The guidelines for the recommendation of antiviral drugs are unsure. The favorite and most commonly used drug of Chinese doctors is mecobalamin. Disappointingly, the use of mecobalamin statistics alone does not achieve the same good results as the use of antivirals. Some studies and guidelines were recommended that combined treatment maybe a good choice.^[26,27]^ Our study showed that the use of steroids plus antivirals and the use of steroids plus mecobalamin are 2 good choices.

Until now, although most of Chinese doctors have not read the guidelines, almost patients with BP can recovery in a high proportion. However, for severe patients and those with hindrance to recovery factors, there is still a lack of effective treatments. More rational prospective studies are needed to further explore the optimization of treatment. The effect of acupuncture also needs to be further evaluated, so that we may have more ways when we encounter those complicated patients.

### 4.1 Limitations

There are some limitations of this study that should be discussed. First, this is a retrospective study. The use of drugs doesn’t follow the guidelines, and more is the clinical experience of doctors. Second, we can only understand the patient's condition and the clinical outcome according to the records of the electronic medical records, and the doctors occasionally exaggerate the effect of the treatment. Third, early use of acupuncture does not seem to be suitable for acute phase, and we have observed this phenomenon clinically, so we recommend stopping using it for almost all hospitalized patients. However, it is uncertain if the adverse consequences are caused by discontinuation or use of acupuncture.

## 5 Conclusions

BP is a common disease in Chinese patients. Risk factors for bad outcome at baseline included: long patient delay, combined disease, and early use of acupuncture. For the therapeutic factors, use of antivirals, mecobalamin, and acupuncture have negative effect for good outcome. The results showed that the early use of steroids was the only beneficial factor adjusted by the binary logistic regression model, however, early use of acupuncture and long patient delay were negative factors. Subgroups analysis showed that the programs of the use of steroids plus antivirals and the use of steroids plus mecobalamin were all good choices. For drug dose and treatment course, long time use of steroids didn’t mean good outcome. Finally, the follow-up showed the improved patients at discharge had better results finally.

## Acknowledgments

The authors thank all the patients who were followed up in the study. They thank Dr Zhigang Chen and Dr Xin He, coming from the Second Affiliated Hospital of Zhejiang University, for their assistance with research design and data processing.

## Author contributions

**Data curation:** Haixia Du, Shenghua Zhou, Jing Chen.

**Formal analysis:** Mingjing Qian.

**Funding acquisition:** Haitong Wan, Jiehong Yang.

**Investigation:** Yu Wang.

**Methodology:** Yu Wang, Shenghua Zhou, Jing Chen.

**Project administration:** Yu Wang.

**Resources:** Hongbo Zhang, Mingjing Qian, Shenghua Zhou, Jing Chen.

**Software:** Haixia Du, Mingjing Qian.

**Supervision:** Haitong Wan, Jiehong Yang.

**Validation:** Jiehong Yang.

**Visualization:** Jiehong Yang.

**Writing – original draft:** Hongbo Zhang.

**Writing – review & editing:** Hongbo Zhang, Haixia Du.
